# Effects of Alendronate Sodium Content on the Interface Strengths of Composite Acrylic Bone Cement

**DOI:** 10.1155/2015/502820

**Published:** 2015-07-27

**Authors:** De-Ye Song, Xin-Zhan Mao, Mu-liang Ding, Jiang-Dong Ni

**Affiliations:** Department of Orthopaedics, 2nd Xiangya Hospital, Central South University, Changsha, Hunan 410011, China

## Abstract

*Objective*. Aim to study how the content of alendronate affected shear strengths at bone-bone cement-metal interfaces.* Methods*. All samples were divided into 6 groups, G_0_–G_5_. On the 1st and 60th day after surgery, bone-bone cement interface shear strengths and bone densities were examined. Interface strengths of metal-bone cement specimens were studied before immersion and 4 weeks after immersion.* Results*. On the 60th day, bone-bone cement interface shear strengths and bone densities showed significant differences (*P* < 0.05), and compared with G_0_, G_2_–G_5_ values increased significantly (*P* < 0.05), and the peak value was met in G_3_. Compared with the 1st day, on the 60th postoperative day both factors decreased significantly in G_0_ and G_1_ (*P* < 0.05). Four weeks after immersion, with the increasing dose of alendronate, the shear strengths decreased gradually and in G_5_ decreased significantly (*P* < 0.05). Compared with before immersion, the metal-bone cement interface strengths decreased significantly 4 weeks after immersion (*P* < 0.05).* Conclusions*. 50–500 mg alendronate in 50 g cement powders could prevent the decrease of shear strengths at bone-bone cement interfaces and had no effect on metal-bone cement interface strengths. While the addition dose was 100 mg, bone cement showed the best strengths.

## 1. Introduction

With the growing aging population, the number of osteoporotic elderly hip fracture patients has been gradually increasing [1]. Bone cement prosthesis replacement has become a very effective method to treat these fractures [2]. Due to the continual friction between the joint prosthesis, aseptic loosening induced by wear particles has become the main reason of the failure in long-term joint replacement [3]. Therefore, how to prevent bone loss and the aseptic loosening after joint prosthesis replacement has become a research focus.

As a class of synthetic analogs of pyrophosphate, bisphosphonate is a potent new drug to inhibit bone resorption [4]. Experiment researches had shown that the drug could inhibit bone loss after joint prosthesis arthroplasty [5], continuously increasing bone densities around the prostheses [6], inhibiting the release of osteolytic factors [7], inhibiting osteolysis induced by wear particles [8], promoting the proliferation and differentiation of osteoblasts [9], enhancing osteoblast activity, inhibiting apoptosis of osteoblasts [10] and bone absorption of osteoclasts, and accelerating apoptosis of osteoclasts [11]. It is supposed that the drug may be an ideal drug to prevent and cure aseptic loosening after prosthesis replacement.

Oral taking is a major administration of bisphosphonates. If these drugs are taken orally for a long time, they have a lot of side effects, such as low bioavailability, high treatment costs, and upper gastrointestinal ulcers [12, 13]. To avoid these side effects, the topical use added in acrylic bone cement may be a better way of administration. Alendronate is a third generation of bisphosphonate and a regular drug used in the treatment of osteoporosis and osteoporotic fractures. In the form of powder, it has some advantages of being mixed easily in bone cement powders, high temperature resistance and remaining drug efficacy in bone cement, and so forth. Literatures reported that acrylic bone cement compounded with alendronate had a favorable biocompatibility [14], and certain contents of alendronate showed no detrimental effects on the fatigue life of composite acrylic bone cement [15].

Bone-bone cement and metal-bone cement interfaces are common sites of aseptic loosening after bone cement joint replacement. However, there is no report about whether these interfaces of composite bone cement being affected or not when alendronate is added. To this end, we used composite acrylic bone cement with different dose of alendronate and made the research mentioned above. The aim was to investigate the change of interface strengths, bone densities, and interface microstructure after alendronate was added.

## 2. Materials and Methods

### 2.1. Experimental Animals and Materials

**Figure 1:**
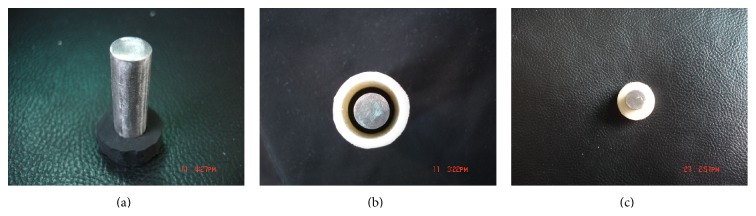
(a) Stainless steel cylinders locating in the center of the axial positioning ring; (b) The metal rod positioning system: stainless steel cylinders locating in the center of hollow polypropylene tubes; (c) Metal-bone cement specimens.

Pure alendronate powder (Merck, USA) and Cemex XL bone cement (Tecres SpA, Verona, Italy) were used as received; stainless steel cylinders (diameter 10 mm × length 37 mm), hollow polypropylene tube (inside diameter 16 mm × outside diameter 20 mm × height 20 mm), the axial positioning ring (Figure 1(a)) (inside diameter 10 mm × outside diameter 20 mm × thickness 10 mm), the metal rod positioning system (Figure 1(b)), and the universal tester (type INSTRON 8032) were obtained from Institute of Biological Materials, Central South University. New Zealand rabbits were supplied by the animal Laboratory, the Second Xiang'ya Hospital. The bone density scanner was supplied by the endocrine laboratory of the Second Xiang'ya Hospital. The study design and experimental procedures were approved by our institution's Animal Care and Use Committee.

### 2.2. Grouping

According to the amount of alendronate added, all drug samples were divided into 6 groups, G_0_–G_5_ (i.e., 0, 10, 50, 100, 500, and 1000 mg alendronate were added in 50 g bone cement powder, resp.).

### 2.3. Preparation of Metal-Bone Cement Interface Strength Specimens

Bone cement was mixed with different dose of alendronate according to the dose regimes above. Then the full reacted mixture was injected into hollow polypropylene tubes, respectively. Stainless steel cylinders with positioning rings were slowly inserted into these tubes. The positioning rings were adjusted to their outside diameter overlapping with the outside diameter of the pipes. After bone cement had solidified, the positioning rings were removed, and bone cement-metal interface specimens were prepared (Figure 1(c)).

### 2.4. Measurement of Metal-Bone Cement Interface Shear Strengths

Specimens were placed on the INSTRON 8032 universal tester, and ten specimens per group, five specimens before immersion, and five specimens 4 weeks after immersion were tested. The metal cylinders were pushed out at the speed of 5.0 mm/min, and the maximum force launched (*F*) was measured. The metal-bone cement interface shear strengths (*E*) were calculated by the following formula and its units were MPa. Consider

(1) $$\begin{array}{c} E=\frac{F}{\pi \cdot d\cdot h}. \end{array}$$

In the equation: *F* stand for metal cylinder's maximum force launched, in the unit of Newton (N), *d* for metal cylinder's diameter (10 mm), and *h* for the height of metal off the bone cement interface (20 mm).

### 2.5. Microscopic Observation of Metal-Bone Cement Interfaces

Six specimens examined by push-out test were chosen (one specimen before immersion and one specimen 4 weeks after immersion for G_0_, G_3_, and G_5_). These samples were cut longitudinally into four equal parts by electric saws. One part of samples was coated with gold and these interfaces were observed by electron microscopy.

### 2.6. Preparation of Bone-Bone Cement Interface Shear Strength Specimens

New Zealand rabbits were operated under intraperitoneal anesthesia (1% sodium pentobarbital, 1.5–2.0 mL/kg). After the success of anesthesia, the surgical area was shaved and cleansed well with 5% benzalkonium bromide and draped the operation area. During the surgery, the rabbits were supplemented with 1% lidocaine as local anaesthetics. An incision about 1.5 cm was made to expose the distal femur by the lateral patellar approach. 3.5 mm drill was used to prepare bone holes and it orientated from the femoral attachment point of the lateral collateral ligament to the femoral medial condyle. When the medial skin of knee was lifted, the incision about 1.0 cm was extended to expose the medial condyle. The wound and bone tunnel were repeatedly washed with hydrogen peroxide and saline, and hemostasis was achieved with fine gauze. Bone cement liquid monomer was mixed with its powders containing different amounts of alendronate. When the reaction was full, the mixture was filled into a volume of 20 mL injector and injected into the bone tunnels in the bilateral distal femurs. Moderate pressure was applied to both ends of the tunnel until the bone cement solidified. The wound was washed twice and closed layer by layer. Penicillin (800,000 U) was injected by intramuscular injection every day after surgery for 7 days. During the observation period, the rabbits were fed under a standard diet and raised in separate cages.

### 2.7. Preparation of the Bone-Bone Cement Interface Specimens

Twelve rabbits were assigned to each group. Six rabbits were sacrificed on the 1st day, and the other six rabbits on 60th day after surgery. The lower ends of the femurs, which contained the specimens, were removed. The left specimens were used to test the bone-bone cement interface shear strengths, while the right ones were used to scan bone densities surrounding the interfaces.

### 2.8. Test of Shear Strength at the Bone-Bone Cement Interfaces

Bilateral femur condyles of the rabbits were trimmed to bone-bone cement interface samples with 9.0 mm lengths with scalpel. Then the specimens were loaded on an INSTRON 8032 universal tester, with a loading speed of 5 mm/min. The tester was halted until the load began to decline gradually. The maximum load was recorded, and the interface shear strengths (*E*), in the unit of MPa, were calculated by the following equation:

(2) $$\begin{array}{c} E=\frac{F}{\pi \cdot d\cdot l}. \end{array}$$

In the equation: *F* stand for maximum load force launched, in the unit of Newton (N), *d* for bone cement cylinder's diameter (3.5 mm), and *l* for the length of bone-bone cement interface (9.0 mm).

### 2.9. Bone Densities Surrounding the Bone-Bone Cement Interfaces

Specimens were trimmed to bone-bone cement interface with 3.0 mm thickness with scalpel. Then cut unnecessary bone and make these samples of a standard size (length 6.0 mm × width 6.0 mm × thickness 3.0 mm) and at the same time make sure the bone cement cylinders locate in the center of the specimens. Specimens were scanned using a bone density scanner and the bone densities were calculated by the tester's software.

### 2.10. Statistical Analysis

SPSS 13.0 for Windows software was used for the statistical analysis. Each set of data were expressed as mean ± standard deviation (SD) and one-way analysis of variance was performed. If there was a significant difference, pairwise comparison was carried out between groups using Scheffe post hoc test. Paired *t*-tests were used for the shear strengths and the bone densities at bone-bone cement interfaces between the 1st day and 60th day after surgery, and metal-bone cement interface strengths between before immersion and 4 weeks after immersion. Test level bilateral *α* = 0.05 and *P* < 0.05 was considered statistically significant.

## 3. Results

### 3.1. Shear Strengths of the Bone-Bone Cement Interfaces

Table 1 showed that on the 1st day after surgery, bone-bone cement interface shear strengths showed no significant differences in all groups (*P* > 0.05). However, on the 60th day after surgery, they showed significant differences (*P* < 0.05), and compared with G_0_, bone-bone cement interface shear strengths in G_1_ showed no significant differences (*P* > 0.05), but in the other groups their values increased significantly (*P* < 0.05), and the peak value was met in G_3_. Compared with the 1st postoperative day, on the 60th postoperative day bone-bone cement interface shear strengths significantly decreased in G_0_ and G_1_ (*P* < 0.05), not in G_2_ to G_5_ (*P* > 0.05).

### 3.2. Bone Densities Surrounding the Bone-Bone Cement Interfaces

Table 2 showed that on the 1st day after surgery, bone densities surrounding bone-bone cement interfaces showed no significant differences in all groups (*P* > 0.05). However, on the 60th day after surgery, they showed significant differences (*P* < 0.05), and compared with G_0_, bone-bone cement interface shear strengths in G_1_ showed no significant differences (*P* > 0.05), but in the other groups their values increased significantly (*P* < 0.05), and the peak value was met in G_3_. Compared with the 1st postoperative day, on the 60th postoperative day bone densities surrounding bone-bone cement interfaces significantly decreased in G_0_ and G_1_ (*P* < 0.05), not in G_2_ to G_5_ (*P* > 0.05).

### 3.3. Shear Strengths of Metal-Bone Cement Interfaces

Table 3 showed that before immersion, metal-bone cement interface strengths in all groups showed no significant difference (*P* > 0.05), while 4 weeks after immersion, with the increasing dose of alendronate, the shear strengths decreased gradually, and in G_5_ the decrease showed significant difference (*P* < 0.05). Compared with that before immersion, the metal-bone cement interface strengths significantly decreased in all groups 4 weeks after immersion (*P* < 0.05).

### 3.4. Electron Microscopy Observation of the Metal-Bone Cement Interfaces

**Figure 2:**
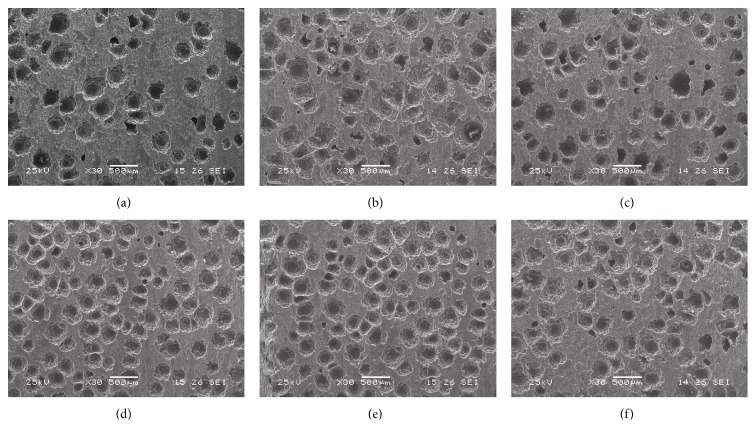
Electron microscopy of the metal-bone cement interfaces: (a) and (d) before immersion and 4 weeks after immersion in G_0_, respectively; (b) and (e) before immersion and 4 weeks after immersion in G_3_, respectively; and (c) and (f) before immersion and 4 weeks after immersion in G_5_, respectively.

Figure 2 showed that the porosity of bone cement specimens was similar before immersion, or 4 weeks after immersion in G_0_, G_3_, or G_5_. But compared with before immersion, the porosity in the same group increased obviously 4 weeks after immersion.

## 4. Discussion

The interfaces between the femoral component and bone cement were known to be a weak area of bone-bone cement prosthesis complex [16]. Previously, Harris and Jasty found that the main mechanism of aseptic loosening on the femoral side was the debonding of the femoral component-bone cement by analyzing the prosthesis removed. Finite element analysis showed that shear stress was a major stress factor for joint prosthesis failure [17]. Interface shear strengths were influenced by a variety of factors, including surface roughness of the femoral stem component, preheating or precoating of the stem component [18], precooling of bone cement monomer, the type of bone cement, the type of prosthesis metal, and the load rate. As a part of this study, we investigated the effects of alendronate on metal-bone cement interface shear strengths. The results showed that before immersion, there is no significant difference in the metal-cement interface strengths in all groups, while 4 weeks after immersion, with the increasing dose of alendronate, the shear strengths decreased gradually, and in group G_5_ the decrease showed significant difference. Meanwhile, electron microscopy showed that no significant difference was found with regard to the interface porosity before immersion or 4 weeks after immersion in groups G_0_, G_3_, and G_5_. These results indicated that the decrease of shear strength of metal-bone cement, was more attributed to decrease of the bone cement bonding capacity than the interface porosity. These studies also found that, compared with that before immersion, 4 weeks after immersion the metal-bone cement interface strengths decreased significantly in the same group. Therefore, it was proposed that the main factor decreasing bone cement bonding capacity might be related to immersion in saline.

Bone-bone cement interfaces were another common site of aseptic loosening after joint prosthesis replacement. According to published reports, aseptic loosening about approximately 50–79% was found 15 years after total hip arthroplasty for young active patients, and 16% of these patients needed revision arthroplasty [19]. Because of the impossible bonding between hydrophobic bone cement and hydrophilic bone tissues, bone cement was used as fillers instead of binders [19]. Instead, interfaces between bone cement and bone tissue become stable fixation by a mechanical intercourse locking. Some studies showed that the shear strengths of bone-bone cement interfaces could be increased by enhancing microlocking between bone cement and bone, and precoating with a layer of an amphiphilic substance on the bone surface. There are many factors that can influence the interface shear strengths, including bone porosity [20], trabecular orientation [20], continuous pressures on the cement [20], preparation of bone surface, and viscosity of bone cement. Moran et al. found that shear strengths of bone-bone cement interfaces were not influenced by gentamicin (0.5 g, 1.0 g, 2.0 g, or 4.0 g) added in 40 g bone cement powders [21]. Moreover, the shear strengths in bone cement were higher than that at bone-bone cement interfaces. Therefore, it is obvious that shear strengths of bone-bone cement interface are a key factor for joint prosthesis service life.

This study found that on the 1st day after surgery shear strengths of bone-bone cement interface in all groups showed no significant difference. However, significant differences were observed on the 60th day after operation. Compared with the 1st day, the interface strengths decreased significantly after 60 days after surgery in G_0_ and G_1_, but no obvious changes were shown in G_2_–G_5_. To investigate the reason of shear strengths' changes, we scanned the bone densities surrounding the bone-bone cement interfaces on the 1st and 60th day after surgery. The results showed that there were similar changes between bone densities and shear strengths at bone-bone cement interfaces. According to the phenomenon above, we inferred that the bone densities might be an important factor to decide the shear strengths at bone-bone cement interfaces. The reason was that bone cement had better mechanical strengths than trabecular bone tissue. Meanwhile, in G_0_ and G_1_, bone densities had significant reduction after 60 days after operation. It might be related to surgical trauma, thermal damage from bone cement, and activities reduction of rabbits. However, in G_2_–G_5_, bone densities at bone-bone cement interfaces showed no significant change. This might result from mineralization capacity enhancement of osteoblast and function inhibition of osteoclast, which was caused by the topical release of alendronate and offset of the negative effects on bone densities.

The advantages and disadvantages of this study also deserved discussion: (1) In this study, we used distal femurs of New Zealand rabbits to prepare the bone-bone cement interfaces. Compared with diseased femoral heads used by Moran [21], the advantages included convenience of obtaining specimens, and an increase in sample volume, and avoiding negative impact from structure differences in diseased bone. However, using healthy tissue also had some drawbacks. These specimens obtained were smaller and more difficult to prepare bone-bone cement interfaces. When alive specimens subjects were used, the bleeding at the interfaces could affect the study results. (2) As artificial femoral stem substitute, stainless steel cylinder had different morphology and surface friction coefficient, which resulted in different shear strength values. However, we had already homogenized these factors that might affect the interface strengths in our experiments, therefore the study results were reliable.

In conclusion, these results showed that a certain amount of alendronate in bone cement had a remarkable effect on interface strengths of composite acrylic bone cement and interfacial bone densities. A dose of 50–500 mg alendronate in 50 g bone cement powder could prevent the decrease of interface strengths at bone-bone cement interfaces, and it had a similar effect on bone densities around these interfaces. However, the same doses of alendronate showed no effect on the interface strengths of metal-bone cement interfaces before immersion and 4 weeks after immersion. While the addition dose was 100 mg, bone cement showed the best strengths. The results of this study indicated that alendronate-loaded bone cement could be made, but alendronate amount must be controlled to below a certain level which had no effect on the shear strengths at metal-bone cement-bone interfaces.

## Figures and Tables

**Table 1:** Shear strengths of bone-bone cement interfaces (MPa) on the 1st day and 60th day after surgery in each group.

Group	1st day	60th day	*P* ^#^
G_0_	5.5372 ± 0.2516	3.6700 ± 0.1341	<0.05
G_1_	5.5868 ± 0.1729	3.7600 ± 0.1707	<0.05
G_2_	5.5573 ± 0.2041	5.6625 ± 0.2906^*^	>0.05
G_3_	5.5630 ± 0.2708	5.6967 ± 0.2170^*^	>0.05
G_4_	5.6450 ± 0.2843	5.6100 ± 0.2184^*^	>0.05
G_5_	5.5330 ± 0.1787	5.6300 ± 0.1975^*^	>0.05
*P* ^##^	0.981	<0.05	

Note: ^##^indicates one-way analysis of variance (ANOVA); ^#^indicates *t*-test, *P* < 0.05; ^*^indicates Scheffé's post hoc test, *P* < 0.05 compared with G_0_.

**Table 2:** Bone densities surrounding the bone-bone cement interfaces (g/cm^2^) on the 1st day and 60th day after surgery in each group.

Group	1st day	60th day	*P* ^#^
G_0_	0.2396 ± 0.0527	0.1356 ± 0.0274	<0.05
G_1_	0.2455 ± 0.0427	0.1313 ± 0.0095	<0.05
G_2_	0.2512 ± 0.0108	0.2509 ± 0.0275^*^	>0.05
G_3_	0.2525 ± 0.0121	0.2584 ± 0.0206^*^	>0.05
G_4_	0.2546 ± 0.0111	0.2554 ± 0.0245^*^	>0.05
G_5_	0.2546 ± 0.0138	0.2512 ± 0.0139^*^	>0.05
*P* ^##^	0.957	<0.05	

Note: ^##^indicates one-way analysis of variance (ANOVA); ^#^indicates *t*-test, *P* < 0.05; ^*^indicates Scheffé's post hoc test, *P* < 0.05 compared with G_0_.

**Table 3:** Shear strengths of the metal-bone cement interfaces (MPa) before immersion and 4 weeks after immersion in each group.

Group	Before immersion	4 weeks after immersion	*P* ^#^
G_0_	5.746 ± 0.7701	4.244 ± 0.0709	<0.05
G_1_	5.668 ± 0.0864	4.200 ± 0.0632	<0.05
G_2_	5.652 ± 0.0834	4.178 ± 0.0581	<0.05
G_3_	5.598 ± 0.1188	4.172 ± 0.0286	<0.05
G_4_	5.564 ± 0.1250	4.138 ± 0.0835	<0.05
G_5_	5.534 ± 0.1043	3.530 ± 0.0418^*^	<0.05
*P* ^##^	0.053	<0.05	

Note: ^##^indicates one-way analysis of variance (ANOVA); ^#^indicates *t*-test, *P* < 0.05; ^*^indicates Scheffé's post hoc test, *P* < 0.05 compared with G_0_.
